# Local and Regional Processes in Community Assembly

**DOI:** 10.1371/journal.pone.0054580

**Published:** 2013-01-23

**Authors:** Juan C Márquez, Jurek Kolasa

**Affiliations:** Department of Biology, McMaster University, Hamilton, Ontario, Canada; Utah State University, United States of America

## Abstract

Controversy on whether local (deterministic) or regional (stochastic) factors control the structure of communities persists after decades of research. The main reason for why it has not been resolved may lie in the nature of evidence which largely comes from realized natural communities. In such communities assembly history leaves a mark that may support either set of factors. To avoid the confounding effects of assembly history we controlled for these effects experimentally. We created a null community by mixing 17 rock pool communities. We then divided the null community into replicates and distributed among treatments representing a gradient of factors from local to regional. We hypothesized that if deterministic factors dominate the assembly of communities, community structures should show a corresponding gradient from being very similar and convergent to dissimilar and divergent. In contrast, if local processes are predominantly stochastic in nature, such a gradient of community configurations should emerge even in the homogeneous setting. Our results appear to partially support both hypotheses and thus suggest that both deterministic and stochastic processes contribute to the assembly of communities. Furthermore, we found that to satisfactorily explain patterns observed in natural communities environmental heterogeneity and regional processes must also be considered. In conclusion, although deterministic mechanisms seem to be important in the assembly of communities, in natural systems their signal may be diluted and masked whenever other factors exert meaningful influence. Such factors increase the number of possible paths to the point that the number of paths equals the number of communities in a metacommunity.

## Introduction

Although the assembly of communities from the available species pool is generally viewed as due to a mix of stochastic and deterministic patterns, a consensus is lacking as to the sources and effects of these two causal agents. For example, we may want to know if any regularities exist in the contribution of local processes (e.g., competition, environment) versus regional processes (e.g., immigration-emigration) [1]–[3], [4], [5]. Such questions are closely related since local processes are generally seen as deterministic (regulated by species already present and by local environmental conditions) while regional processes are seen as stochastic (identity of potential colonizers is independent of local conditions) [1], [2], [6].

Local assembly invokes niche-based processes as a key mechanism [7], [8], [9], [10], [11]. According to this perspective, a new community forms from species first filtered by their environmental requirements. These species are further filtered according to assembly rules thought to arise from biological interactions [12]–[14]. Since environmental and biological filtering operates regardless of the properties of the regional species pool and the expected outcome is defined by specific conditions and specific species combinations, such processes are seen as deterministic [15], [16]. This proposition is amenable to experimental tests because it allows specific predictions. For example, communities assembling under similar environmental regimes and equally accessible to colonizers should follow a common path if the mechanisms are deterministic. Consequently, such communities should converge in composition and abundance structure.

In contrast, if regional processes are more important than local processes in the assembly of communities, community structure should be predominantly shaped by stochastic processes such as immigration, order of colonization and extinction - all of which depend on the size and nature of the regional pool [6], [17]. As a result, communities developing in a similar environment should be dissimilar.

Difficulties in finding an empirical resolution to these opposing expectations may stem from the fact that studies supporting local or regional processes usually rely on realized communities [18]. Such communities are shaped by multiple factors (e.g., immigration, competition, mutualism, random extinction, local environment [19]–[22] among others) acting during assembly [23]. Consequently, informative patterns of assembly in realized communities are difficult to detect; which makes linking specific mechanisms to the observed community structure difficult [24]. Lengthy discussion on the application of null model analysis to detect patterns from realized communities [14], [25]–[28] exemplifies the problem. Although null models have achieved great sophistication, they still face serious challenges [28]–[30].

On occasions when environment has strong influence (e.g., high disturbance frequency), its signal may be reflected in community structure [31]. Even in such cases however a large proportion of the compositional variability is attributed to history of the assembly process [32]–[34]. In sum, signals due to deterministic mechanisms can be masked by stochastic variation in realized communities and thus make them unsuitable for drawing inferences about their contribution.

We propose to reveal the relative contributions of local and regional processes in the assembly of communities by an experimental strategy. The strategy involves stepwise elimination of confounding influences on assembly. First, we created a null community (a community of aquatic invertebrates formed from a mix of 17 natural communities obtained from rock pools). Because all species were added at the same time, this approach eliminated the history of colonization as a factor. Next, we divided this null community among four treatments comprising 17 to 40 replicate communities. We followed their development for several months. We created four treatments by placing null community in: (a) natural pools with different natural physical and chemical environments, with dispersal allowed and local habitat heterogeneity present; (b) beaker communities that allow physical and chemical external influences but no dispersal; (c) as in treatment ‘b’ plus chemical exchanges, and; (d) as in ‘c’ but placed under the same environmental regime (homogeneous environment). All of these treatments were compared to naturally assembled communities.

We assumed that convergence among community states formed in treatments would be a good indicator of the degree to which deterministic processes shape community structure. Specifically, if local processes are overwhelmingly deterministic, a single community state should emerge in the homogeneous setting (that is all replicate communities should converge and become similar), and progressively divergent states should emerge under treatments that allow other influences (that is dispersal and heterogeneity of conditions to which individual replicates were subjected to). In contrast, if local processes are predominantly stochastic, a gradient of community configurations should emerge in the homogeneous setting - a gradient in which alternative states are difficult to identify. Furthermore, in experimental treatments with more ecological realism, similarity among community replicates (inter-replicate similarity) should be lower than that observed in the homogeneous setting. Finally, if community is structured by both deterministic and stochastic factors, the homogenous setting should produce detectable alternative states with a considerable degree of similarity. With more realistic conditions (remaining treatments), a progressive dilution of these states should be observed. See Table 1 for all the expectations, along with the expectations for the dissimilarity values of the null model. While it is clear that the unobstructed development of communities in the natural setting does not permit discriminating between deterministic and stochastic mechanisms, the decay of their respective signals should follow different paths and thus assist in drawing inferences.

**Table 1:** General outline of treatments and predictions.

				If deterministic mechanisms dominate			Deterministic and stochastic			If stochastic mechanisms dominate		
Treatment	Natural dispersal permitted	Onlyphysical influences permitted	Physicalandchemical influences permitted	Number of differentend states	Inter-replicate similarity(atthe end of experiment)	Beta Raupand Crick dissimilarity index	Number of different end states	Inter-replicate similarity(at the end of experiment)	Beta Raup and Crickdissimilarity index	Number of differentend states	Inter-replicate similarity(atthe end of experiment)	Beta Raup and Crick dissimilarity index
**D. Beakers alone**	−	−	−	One	High	Close to orequal to −1	Few	Low	Higher than −1	None	Low	0
**C. Beakers in** **natural pools**	−	+	−	↓	High but lower than above	Close to −1 but less than above	↓	↓	Higher than −1but more than above	None	↓	0
**B. Altered beakers** **in natural pools**	−	+	+	↓	Inter-mediate	Higher than 0	↓	↓	Slightly higherthan 0	None	↓	0
**A. Natural pool**	+	++	+++	Many	Low	Higher than the above	Many	Very low	Higher than 0	None	Very low	0

General outline of treatments and predictions depending on the assumptions of whether community assembly is dominated by deterministic or stochastic mechanisms. “−“ symbols mean no; “+” symbols mean yes; “++” symbol refers to the fully natural setting where no physical influences or material exchanges are inhibited in any way. Detail description of treatments is given in the Methods section.

## Methods

### Study Site

For the experiments in this study we used an invertebrate community inhabiting a set of rock pools located on the grounds of the University of the West Indies Discovery Bay Marine Lab, Jamaica. No specific permits were required for the described field studies by the institution managing the grounds and no endangered or protected species were involved in our study.

The mean size of the pools is 52 (SD±20 cm) × 30 cm (SD±14 cm), with a mean depth of 12 cm (SD±8 cm) and mean volume of 17 L (SD±18.5 L). Some pools are separated from their neighbors by a few cm and others by several meters. Some of the pools are tidal but the majority are maintained by rain and occasional wave splash water, which produces an diversity of fresh, brackish and salt water pools [35].

The biological community of interest consists of over 70 small benthic and planktonic animals, mostly between 600 µm to 5 mm. The animals represented Turbellaria (7 spp.), Nematoda (1 spp.), Polychaeta (5 spp.), Oligochaeta (2 spp.), Ostracoda (21 spp.), Copepoda (8 spp.), Cladocera (4 spp.), Decapoda (crab) larvae (1 spp.), Decapoda (shrimps) (3 spp.), Amphipoda (1 spp.), Isopoda (1), and Insecta (18 spp.). The life cycle of the animals residing in the rock pools may take from less than one week up to three months [36]. Most communities experience occasional desiccation, especially in the summer and in shallow pools [37].

Previous studies in the rock pool community had identified trophic relationships for some of the species [37], [38]. For example, larger ostracods, like *Candona* sp., are top predators feeding on several trophic levels that range from cladocerans (herbivores) and other ostracods (detritivores) down to detritus. Insects such as mosquitoes feed on phytoplankton, protozoans, and detritus, while beetles and some polychaetes are predatory. Midges however feed also on filamentous algae and detritus.

### Experiments

**Figure 1:**
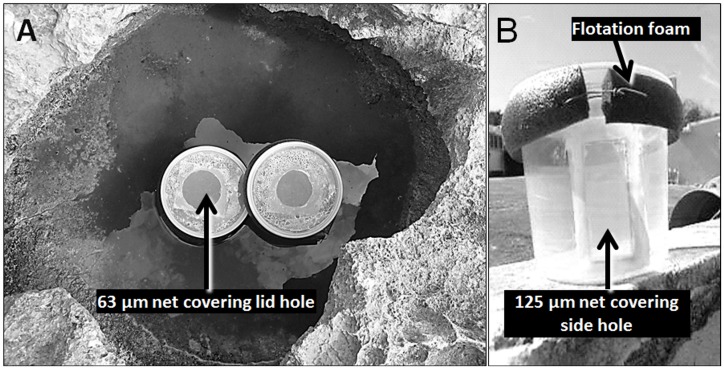
Treatment description. (a) Treatments C (left beaker), B (right beaker), and A (pool); (b) detail of a beaker used for the Treatment B, with a net covered hole for water interchange and foam for floatation.

To implement treatments outlined in Table 1, we first created a null community - a community of all species available in arbitrarily defined region. We created this null community by mixing contents of 17 freshwater (salinity <5 per mil, average volume 10.4 L±SD 7.6) pools that were selected from the set of pools (see Study Site). They were located from one to seven meters apart from the nearest neighbor. Before combining the contents of the pools, we took biotic samples to capture the composition of each individual community. We also measured the volume of water removed from each pool. To kill off any remaining freshwater organisms we cleaned pools thoroughly with seawater, and patted dry with a sponge. We then place the null community into the emptied pools in volumes matching the original volumes (Treatment A). In addition, two beakers were added into each pool (Figure 1a). These beakers were also filled with 400 ml of the null community mix. One of these beakers had a rectangular hole through its side covered by a fine mesh of 125 µm (Figure 1b; Treatment B); the other beaker was not altered (Treatment C). The beakers had a lid opening covered by a net (63 µm mesh size) to exclude airborne invertebrate propagules (Figure 1a). Forty additional beakers were filled up with the null community mix and placed under a similar environmental regime (outdoors, on a bench; Treatment D). Treatment A served to assess the effects of naturally diversified environments on the community development without differences due to initial colonization success. Treatment B served to evaluate the effect of local physical and chemical conditions in the absence of the influence of colonization history, subsequent dispersal, or habitat heterogeneity (due to morphometric differences among pools). Treatment C aimed to provide further reduction of natural local influences associated with the abiotic and indirect biotic factors. Treatment D aimed to isolate internal community processes from all other factors that might affect developmental trajectories. Table 1 provides an overview of the experimental design in addition to listing theoretical expectations mentioned earlier.

We sampled the null community mix at the beginning of the experiment, before it was distributed among treatments, and each treatment after 4 months. Contents of the beakers and natural pools were homogenized by stirring the water while sampling for invertebrates. Samples consisted in 100 ml of water which was passed through a 63 µm net. Animals retained in the net were transferred into a 50 ml vial with 50% ethanol. In laboratory, organisms were separated using a stereomicroscope following an established visual method in which more than 95% of the individuals are removed from the sample [39], identified and counted.

### Data Analysis

First, we used a null model to detect signals of deterministic, stochastic or intermediate contributions of these processes using the data transformed to presence/absence matrix. Second, we identified alternative states using a similarity measure based on the distribution and abundance of the species. We describe these two steps below.

#### Null model

We used the PaST (Paleontological STatistics) program [40] to obtain the Raup and Crick [41] similarity index. We transformed similarity values to dissimilarity and standardized to range from −1 to 1 following Chase et al. [42] modification. The model randomizes the presence-absence of the species of the regional pool constrained by the frequencies of species in each site (1000 random replicates). The value of the index is based on the number of shared species observed and shared species expected [42] between each pair of sites. The mean value of the resulting dissimilarity matrix is used to compare treatments. Values close to zero indicate randomness; values around −1 indicate communities more similar than expected and positive values indicate communities less similar than expected [42]. We applied this model to the communities observed after four months of the experiment in treatments A, B, C and D as well as to the communities found in natural pools before the mixing.

#### Classification and ordination

Although the null model we applied does a good job in identifying structuring forces in the assembly of communities, it might deliver confusing signals in some cases. Chase et al. [42] admits that the dissimilarity metrics have an “unavoidable” problem when local diversity is very low relative to the species pool, as expected in the beakers with no dispersal. In such situations the number of shared species edges towards zero, decreasing the power of the index to detect deviations from the null expectation [42]. Also, and more importantly, the index might underestimate deterministic signals. For example, in a metacommunity there might be several deterministic paths initiated by the involvement of stochastic processes. This situation could result in several groups of highly similar communities that are significantly different from each other. In such a case the index would underestimate determinism because the high similarity within groups would be diluted by the low similarities between sites belonging to different groups. To overcome this and related problems we checked for the presence of alternative states and calculated the average similarity using species abundances rather than presences only (see Table 1).

In order to assess the number of plausible alternative states and estimate the average similarity among replicates for each treatment we calculated Bray-Curtis similarity index between all possible replicate pairs. Abundance data were fourth root transformed to down weigh high abundance species and allow rare species to contribute more to the overall similarity.

We used similarity values to perform two analyses. First, using a hierarchical cluster analysis (group average as the clustering mode) we grouped samples from each community, regardless of treatment, according to their similarity in species composition and abundance. To identify alternative states within treatments we adopted a stringent group defining criterion. The criterion was the minimum similarity (defined by the least similar replicate of that community) that retained null community samples together as one group. Thus, any group recognized as an alternative state must have contained communities at least as similar as the homogenized null community the experiment had begun with and differ from all other such groups or individual communities. A potential caveat is due to the fact that high similarity among founding communities (e.g., NC controls) leaves a limited parameter space for further increase in similarity. To determine whether the resulting alternative states were significantly different and not an artifact of the high similarity threshold used to identify them, we used an analysis of similarities (ANOSIM protocol in PRIMER 6).

The second analysis involved a combination of procedures to visualize the degree of convergence and divergence between groups of communities (alternative states). To accomplish this, we first applied a non-metric multi-dimensional scaling (NMDS) to the Bray-Curtis similarity matrix. Then, we superimposed the community classification produced by the cluster analysis onto the NMDS graph in order to evaluate the mutual consistency of both representations [43]. Here, alternative states from the cluster analysis appear in the NMDS as ellipses encircling samples (points). Both analyses were performed using PRIMER 6. Finally, we also tested for the significance of the differences in the structure (distribution and abundance of species) between treatments using permutational analysis of multivariate dispersions (PERMDISP2 [44]). This procedure compares groups according to their level of dispersion around a centroid. Finally, because differences among treatments in container volume have significantly affected richness and density of replicate communities, they may have systematically influence within-treatment similarities. To verify that this was not the case, we have conducted an ANOVA analysis where treatments with different volumes were factors. We found that mean similarities did not differ among the treatments (F = 0.002, p = 0.969) and thus did not affect our general findings.

We intentionally avoided characterizing the alternative states as stable or not as such characterization does not seem to contribute to answering our questions, primarily because such state appear transient [45].

## Results

### Null Model

**Figure 2:**
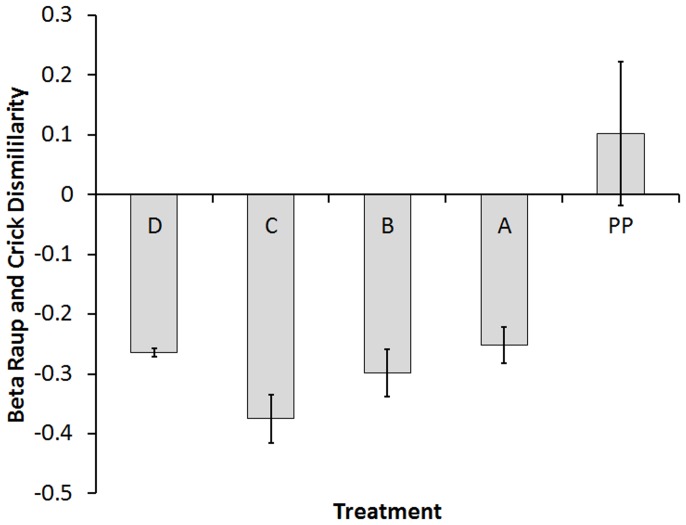
Beta Raup and Crick dissimilarity index. Mean dissimilarity values for each one of the treatments. Whiskers indicate standard error.

Patterns generated by the null model conform to the scenario in which both determinism and stochasticity play a role in the assembly of communities. Deterministic signal was higher than −1 in Treatment C (mean −0.37± standard error 0.04) and became weaker as treatments included more factors. As expected, the highest value of the index was found in the PP treatment (natural pools), indicating an important influence of environment and dispersal in the assembly process. Counter intuitively Treatment D showed a lower level of determinism (−0.26±0.007) when compared to the other treatments, closer to the value showed by the pools with dispersal and environmental differences permitted (Treatment A, −0.25±0.03) (Figure 2). This appears to be due to the weaknesses of this particular null model (see Methods) when local diversity is low and when deterministic signal is diluted (see the section below on alternative community states).

### Similarity of Community States

**Figure 3:**
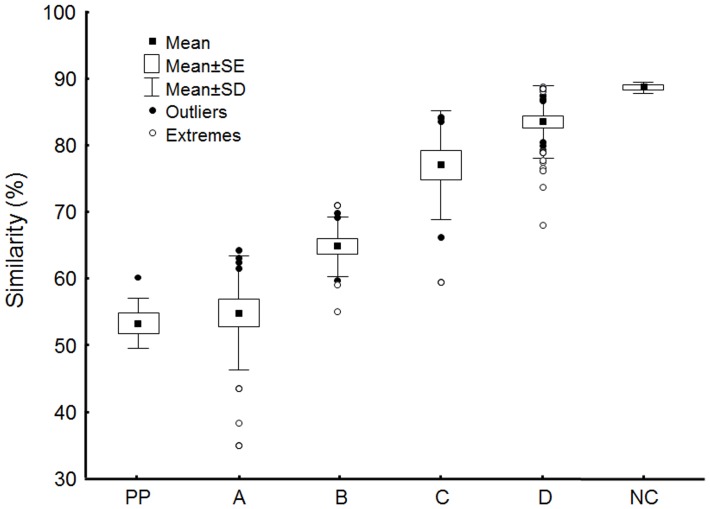
Comparison of mean similarity among treatments. Mean similarity values are based on all communities within a single treatment. PP –pool communities prior to mixing, A - communities in natural pools (after they developed from the null community); B - immersed beaker communities with material interchange permitted but no dispersal; C - immersed beaker communities without dispersal and material interchange, and D - beaker communities in homogeneous setting.

We found that the levels of inter-replicate Bray- Curtis similarity formed a gradient entirely consistent with the expectation in which both set of processes contribute in the assembly (Table 1). Specifically, the null community (NC) samples had the highest similarity (88%, n = 5, standard error (se) ±0.38), communities in natural pools, PP, had the lowest inter-replicate similarity (53%, n = 6, se ±1.55), and the remaining treatments (Treatment A, B and C) showed increasing levels of inter-replicate similarity (Figure 3).

Most of the treatments differed significantly from each other and those that did not (Table 2) are consistent with the general pattern nevertheless. Specifically, closed beaker communities in homogeneous setting (Treatment D) were most similar to the null community (NC). Furthermore, pool communities assessed prior to mixing (PP) had the same level of similarity as communities developed from null community in the same set of natural pools (Treatment A). Internal similarity of Treatments B and C increased gradually as the factors affecting assembly were experimentally reduced. This trend underscores the dominant role of interactions between local and regional processes in natural settings.

**Table 2:** PERMDISP2 test results comparing dispersion between treatments.

Treatment	PP	A	B	C	D	NC
PP						
A	0.85778					
B	0.00567*	0.02497*				
C	0.00508*	0.00143*	0.07346			
D	0.00000*	0.00000*	0.00000*	0.01677*		
NC	0.00002*	0.00017*	0.00000*	0.03822*	0.27201	

PERMDISP2 test results comparing dispersion between treatments. Numbers are p-values and asterisks indicate significant differences.

### Alternative Community States

**Figure 4:**
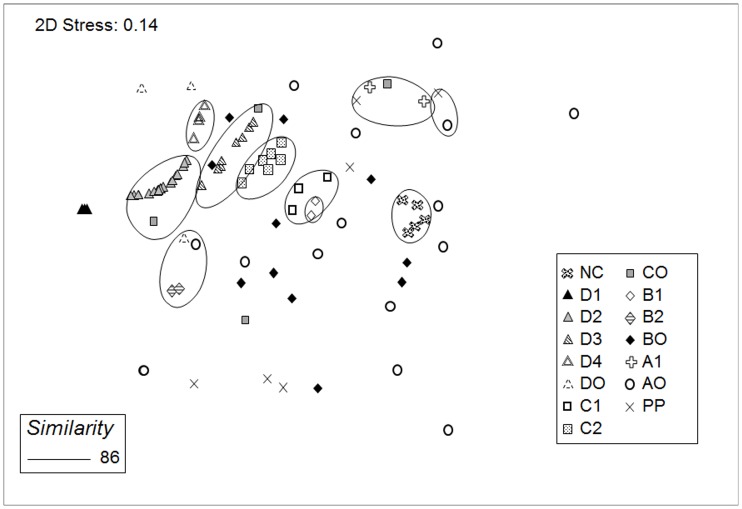
Alternative states within treatments. Alternative states (ellipses) formed at the 86% similarity level (see explanation in text) for each of the treatments. Stress values <0.2 indicate an acceptable (significant) ordination. Horizontal axis represents NMDS dimension 1 and vertical axis represents NMDS dimension 2.

The trend in the number of alternative states also corresponds with our hypothesis (Table 1): The more factors involved in the assembly, the higher the number of alternative states. Significantly, alternative states emerge even in the most homogenous conditions (Treatment D). In that treatment we found four such states represented by more than one community (i.e., collective alternative states, CAS), and 3 unitary alternative states (i.e., alternative states with one member, UAS). The next most isolated group (Treatment C) formed two CAS, and four UAS. Treatment B communities formed two CAS, each with two members, with the remaining communities (11) representing UAS. Within Treatment A only one alternative state with two members was detected. The remaining communities (15) were UAS. Communities in the natural pools, PP) failed to form identifiable CAS. Consequently, all replicates fell into the UAS category (Figure 4).

Differences among CAS were significant, between and within treatments (Table 3) except when the number of permutations was low, that is, when the two groups compared had two or, exceptionally, three members.

**Table 3:** ANOSIM results for the pairwise comparisons of the similarity among alternative states.

Alternative State	D1 (n = 3)	D2 (n = 21)	D3 (n = 8)	D4 (n = 4)	C1 (n = 3)	C2 (n = 7)	B1 (n = 2)	B2 (n = 2)		A1 (n = 2)
**D1**										
**D2**	0.020*									
**D3**	0.006*	0.001*								
**D4**	0.029*	0.002*	0.002*							
**C1**	0.1	0.002*	0.006*	0.029*						
**C2**	0.008*	0.001*	0.001*	0.003*	0.008*					
**B1**	0.1	0.004*	0.022*	0.067	0.1	0.028*				
**B2**	0.429	0.004*	0.0022*	0.067	0.1	0.028*	0.33			
**A1**	0.1	0.004*	0.0022*	0.067	0.1	0.028*	0.33	0.33		

ANOSIM results for the pairwise comparisons of the similarity among alternative states. Numbers are p-values and asterisks indicate significant differences.

### Other Community Trends

**Figure 5:**
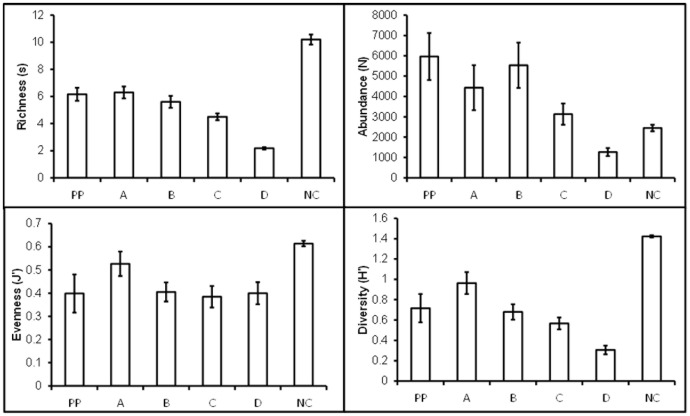
Changes in mean richness, abundance, evenness, and diversity through time for each treatment. State of communities in different treatments at the end of experiment (4 months): mean richness, abundance, evenness, and diversity (bars) and standard error (whiskers) shown.

On average, replicate community abundances increased over time with the exception of the most isolated communities (Treatment D) (Figure 5). By contrast, richness, diversity, and evenness declined across all the treatments (Figure 5).

**Figure 6:**
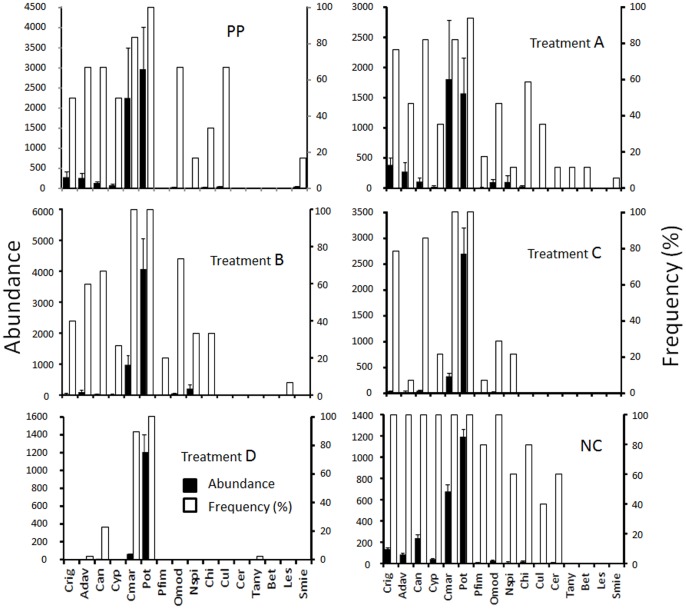
Mean abundance and frequency of species in each treatment. PP – pre-mixed pool communities, Treatment A - communities in natural pools; Treatment B - immersed beaker communities with material interchange permitted; Treatment C - immersed beaker communities without migration and material interchange, and Treatment D - communities in homogeneous setting.

The composition and abundance of species changed differently in various treatments. Individual species either increased or decreased their relative abundance and frequency compared to NC (Figure 6). A total of 16 invertebrate species were identified in the null community samples. Most of the individuals were crustaceans: one decapod (larvae) *Sesarma miersi* (Rathbun); two cladocerans, *Ceriodaphnia rigaudi* (Richard) and *Alona davidii* (Richard); three copepods, *Paracyclops fimbriatus* (Fisher), *Orthocyclops modestus* (Herrick) and *Nitocra spinipes* Boeck; and four ostracods, *Candona* sp., *Cypricercus* sp., *Cypridopsis* cf. *mariae* Rome, and *Potamocypris* sp. The rest of the species where insect larvae represented by three midges: a chiromid, a tanypodid and a ceratopogonid; two mosquitoes: *Culex* sp. and *Anopheles sp.*, and a coleopteran (larvae).

The most abundant of these species were the ostracods *Potamocypris* sp. and *Cypridopsis* cf. *mariae* followed by the cladocerans *C. rigaudi* and *A. davidii*, and ostracod *Candona* sp. Insect larvae and larvae of the crab *S. miersii* were the less abundant. In general, more abundant species showed higher frequencies but both frequency and abundance were more variable in less abundant species (Figure 6).

We did not detect any systematic change in the community trophic structure during the course of the experiment. This is possibly because most of the organisms used in the experiment are opportunistic detritivores or omnivores.

## Discussion

Communities that formed on a gradient of processes from local to regional scale showed a corresponding gradient of declining similarity and of increasing number alternative states. The formation of alternative states (groups of replicates with high similarity at the end of the experiment) in treatments where only local processes were involved suggests that both stochastic and deterministic forces must be in play. The latter further implies that community-wide assembly rules are involved to some extent as some have suggested [46], [47]. This inference is further backed up by a negative, but far from −1, Raup and Crick dissimilarity value for treatment D, which indicates some involvement of stochastic processes during assembly.

The emergence of several community states in a homogeneous environment emphasizes that significant community differences can arise without the interference of community history (commonly discussed in terms of the order of colonization or priority effects). This observation could explain instances where priority effects were detected and other instances where they were not [48], [49]. A tentative conclusion is thus that priority effects are not a requirement for the formation of alternative states even if they may often be involved.

It seems then that assembly rules resulting from the filtering effect during niche accommodation among arriving and constituent species are an influential force in the assembly of communities. This influence however is modified by stochastic events that cause the formation of several deterministic paths among initially similar communities. In the case of treatment D, this might have been caused by demographic stochasticity or simply by small initial differences inevitable during the allocation of the null community among the beakers. Other examples of possible mechanisms include intra-guild predation [50], random extinction of top predators [51] or size-dependent predation by top predators.

Local environmental factors contribute further to the stochastic signal. The structure of communities is often linked to environmental local factors [23], [52], [53], especially among ephemeral ecosystems like some in this study [54]. The number of alternative states observed in Treatment C was higher than in Treatment D when differences in the number of samples are accounted for (Treatment C, 17 beakers; Treatment D, 40 beakers). As Treatment C allows environmental variables to influence communities more than Treatment D, we attribute the increased community divergence (more states, less similarity) to the direct or indirect action of the environment [32], [33], [52], [53]. Determinism may also play a role here. However, because Raup and Crick dissimilarity index was near zero, we must conclude that a strong stochastic component dominated. Buenau et al. [55] found that competing populations inhabiting local communities that differ in environmental conditions can form alternative states. In such situations environmental feedback controls the recruitment success of competing populations as a function of differences in their fitness due to specific local conditions.

Disturbance may have also contributed to differentiation among replicates. Instances of pool desiccation are not uncommon in the studied system [37]. This source of heterogeneity, combined with demographic stochasticity and environmental differences already present, provides a reasonable explanation for a higher number of alternative states and low inter-replicate similarity (and thus close to zero Raup and Crick dissimilarity) among replicates in Treatments B and C.

Although biological interactions and environment accounted for much of the variation involved in the assembly of the experimental communities, it was not until regional processes (Treatment A) were included before the general patterns observed in the natural pools (Pools pre-mixed, PP) were reproduced. This is a clear indication of the importance of dispersal in the assembly processes, although not as strong as suggested by the neutral theory [17]. In this treatment (A), as in others, Raup and Crick dissimilatiy index was negative although closer to zero, which indicates a non-negligible influence of deterministic processes.

All of the above suggests that local, biological and environmental processes, together with regional processes are needed to explain patterns and dynamics in realized communities. Our results give support to the contemporary view in community assembly in which both local and regional processes are important when explaining community structure [56]. According to this view local and regional processes are the extremes of a continuum [57], with local processes and regional processes making different contributions to the final community state [58]. In short, regional processes alone are insufficient to generate the level of structural diversification observed in natural communities.

In conclusion, although deterministic mechanisms seem to be an important factor in the assembly of communities, in natural systems their signal may be diluted and masked whenever other factors exert meaningful influence. Such factors increase the number of possible paths to the point that the number of paths equals the number of communities in a metacommunity. This logic is further corroborated by the totality of results obtained from the remaining treatments: gradual addition of various external influences leads to a complete breakdown of tendency to form similar communities.

## References

[1] Jenkins DG, Buikema AL Jr (1998) Do similar communities develop in similar sites? A test with zooplankton structure and function. Ecol Monogr 68: 421–443.

[2] Jenkins DG (2006) In search of quorum effects in metacommunity structure: Species co-occurrence analyses. Ecol 87: 1523–1531. 10.1890/0012-9658(2006)87[1523:isoqei]2.0.co;2 16869428

[3] Shurin JB (2000) Dispersal limitation, invasion resistance, and the structure of pond zooplankton communities. Ecol 81: 3074–3086.

[4] Shurin JB, Havel JE, Leibold MA, Pinel-Alloul B (2000) Local and regional zooplankton species richness: A scale-independent test for saturation. Ecol 81: 3062–3073.

[5] Ricklefs RE (2008) Disintegration of the ecological community. Amer Nat 172: 741–750. 18954264 10.1086/593002

[6] Hubbell SP (2006) Neutral theory and the evolution of ecological equivalence. Ecol 87: 1387–1398. 10.1890/0012-9658(2006)87[1387:ntateo]2.0.co;2 16869413

[7] Fox BJ, Brown JH (1995) Reaffirming the validity of the assembly pale for functional groups or guilds - Reply. Oikos 73: 125–132.

[8] Fox BJ, Fox MD (2000) Factors determining mammal species richness on habitat islands and isolates: Habitat diversity, disturbance, species interactions and guild assembly rSules. Global Ecol Biogeogr 9: 19–37.

[9] Brown JH, Kelt DA, Fox BJ (2002) Assembly rules and competition in desert rodents. Amer Nat 160: 815–818. 18707468 10.1086/343882

[10] Fargione J, Brown CS, Tilman D (2003) Community assembly and invasion: An experimental test of neutral versus niche processes. Proc Natl Acad Sci USA 100: 8916–8920. 12843401 10.1073/pnas.1033107100 PMC166413

[11] Chase JM (2005) Towards a really unified theory for metacommunities. Funct Ecol 19: 182–186.

[12] Fox BJ (1987) Species assembly and the evolution of community structure. Evol Ecol 1: 201–213.

[13] Belyea LR (2004) Beyond Ecological Filters: Feedback networks in the assembly and restoration of community structure. In: Temperton VM, Hobbs RJ, Nuttle T, Halle S, editors. Assembly rules and restoration ecology: Bridging the gap between theory and practice. Island Press. 115–131.

[14] Diamond JM (1975) Assembly of species communities. In: Cody ML, Diamond JM, editors. Ecology and evolution of communities. Cambridge: Belknap Press of Harvard University Press. 342–444.

[15] Chase JM (2003) Community assembly: when should history matter? Oecologia 136: 489–498. 12836009 10.1007/s00442-003-1311-7

[16] Chase JM, Amarasekare P, Cottenie K, Gonzalez A, Holt RD, et al. (2005) Competing theories for competitive metacommunities. In: Holyoak M, Leibold MA, Holt RD, editors. Mecommunities: Spatial Dynamics and Ecological Communities. Chicago: The Uninversity of Chicago Press.

[17] Hu XS, He FL, Hubbell SP (2007) Species diversity in local neutral communities. Amer Nat 170: 844–853. 18171167 10.1086/522935

[18] Mouquet N, Munguia P, Kneitel JM, Miller TE (2003) Community assembly time and the relationship between local and regional species richness. Oikos 103: 618–626.

[19] Mutshinda CM, O'Hara RB, Woiwod IP (2009) What drives community dynamics? Proc R Soc B Biol Sci 276: 2923–2929. 10.1098/rspb.2009.0523 PMC2817208 19457887

[20] Thompson R, Townsend C (2006) A truce with neutral theory: local deterministic factors, species traits and dispersal limitation together determine patterns of diversity in stream invertebrates. J Anim Ecol 75: 476–484. 16638000 10.1111/j.1365-2656.2006.01068.x

[21] Mouquet N, Miller E, Daufresne T, Kneitel M (2006) Consequences of varying regional heterogeneity in source-sink metacommunities. Oikos 113: 481–488.

[22] Cadotte MW (2006) Dispersal and species diversity: A meta-analysis. Amer Nat 167: 913–924. 16649154 10.1086/504850

[23] Fukami T, Bezemer TM, Mortimer SR, van der Putten WH (2005) Species divergence and trait convergence in experimental plant community assembly. Ecol Lett 8: 1283–1290.

[24] Simberloff D (2004) Community ecology: Is it time to move on? (An American Society of Naturalists Presidential Address). Amer Nat 163: 787–799. 15266378 10.1086/420777

[25] Diamond JM, Gilpin ME (1982) Examination of the null model of Connor and Simberloff for species co-occurrences on Islands. Oecologia 52: 64–74. 28310110 10.1007/BF00349013

[26] Gotelli NJ, Mccabe DJ (2002) Species co-occurrence: A meta-analysis of J. M. Diamond's assembly rules model. Ecol 83: 2091–2096.

[27] Gotelli NJ, Buckley NJ, Wiens JA (1997) Co-occurrence of Australian land birds: Diamond's assembly rules revisited. Oikos 80: 311–324.

[28] Ulrich W (2004) Species co-occurrences and neutral models: reassessing J. M. Diamond's assembly rules. Oikos 107: 603–609.

[29] Ulrich W, Gotelli NJ (2007) Null model analysis of species nestedness patterns. Ecol 88: 1824–1831. 10.1890/06-1208.1 17645028

[30] Gotelli NJ, Ulrich W (2012) Statistical challenges in null model analysis. Oikos 121: 171–180.

[31] Chase JM (2007) Drought mediates the importance of stochastic community assembly. Proc Natl Acad Sci USA 104: 17430–17434. 17942690 10.1073/pnas.0704350104 PMC2077273

[32] Cottenie K, Michels E, Nuytten N, De Meester L (2003) Zooplankton metacommunity structure regional vs. local processes in highly interconnected ponds. Ecol 84: 991–1000.

[33] Cottenie K (2005) Integrating environmental and spatial processes in ecological community dynamics. Ecol Lett 8: 1175–1182. 21352441 10.1111/j.1461-0248.2005.00820.x

[34] Van der Gucht K, Cottenie K, Muylaert K, Vloemans N, Cousin S, et al (2007) The power of species sorting: Local factors drive bacterial community composition over a wide range of spatial scales. Proc Natl Acad Sci USA 104: 20404–20409. 18077371 10.1073/pnas.0707200104 PMC2154443

[35] Romanuk TN, Kolasa J (2001) Simplifying the complexity of temporal diversity dynamics: A differentiation approach. Ecoscience 8: 259–263.

[36] Kolasa J, Romanuk TN (2005) Assembly of unequals in the unequal word of a rock pool metacommunity. In: Holyoak M, Leibold MA, Holt RD, editors. Mecommunities: Spatial Dynamics and Ecological Communities. Chicago: The Uninversity of Chicago Press. 212–232.

[37] Therriault TW, Kolasa J (2001) Desiccation frequency reduces species diversity and predictability of community structure in coastal rock pools. Isr J Zool 47: 477–489.

[38] Beisner BE, Romanuk T (2005) Diversity, productivity and invasibility relationships in rock pool food webs. In: De Ruiter PC, Wolters V, Moore JC, editors. Dynamic food webs: Multispecies assemblages, ecosystem development, and environmental change. Elsevier, Inc. 321–333.

[39] Therriault TW (2002) Temporal patterns of diversity, abundance and eveness for invertebrate communities from coastal freshwater and brackish water rock pools. Aquatic Ecol 36: 529–540.

[40] Hammer O, Harper DAT, Ryan PD (2001) PAST: Paleontological statistics software package for education and data analysis. Palaeontologia Electronica 4: 1–9.

[41] Raup D, Crick R (1979) Measurement of faunal similarity in paleontology. Journal of Paleontology 53: 1213–1227.

[42] Chase JM, Kraft NJB, Smith KG, Vellend M, Inouye BD (2011) Using null models to disentangle variation in community dissimilarity from variation in alpha diversity. Ecosphere 2: art24.

[43] Clarke KR, Warwick RM (2001) Change in marine communities: An approach to statistical analysis and interpretation. UK: PRIMER-E: Plymouth.

[44] Anderson MJ (2006) Distance-based tests for homogeneity of multivariate dispersions. Biometrics 62: 245–253. 16542252 10.1111/j.1541-0420.2005.00440.x

[45] Fukami T, Nakajima M (2011) Community assembly: alternative stable states or alternative transient states? Ecol Lett 14: 973–984. 21790934 10.1111/j.1461-0248.2011.01663.x PMC3187870

[46] Drake JA (1991) Community-assembly mechanics and the structure of an experimental species ensemble. Amer Nat 137: 1–26.

[47] Law R, Morton RD (1996) Permanence and the assembly of ecological communities. Ecol 77: 762–775.

[48] Jiang L, Joshi H, Flakes SK, Jung YJ (2011) Alternative community compositional and dynamical states: the dual consequences of assembly history. J Anim Ecol 80: 577–585. 21226710 10.1111/j.1365-2656.2010.01799.x

[49] Tilman D (2004) Niche tradeoffs, neutrality, and community structure: A stochastic theory of resource competition, invasion, and community assembly. Proc Natl Acad Sci USA 101: 10854–10861. 15243158 10.1073/pnas.0403458101 PMC503710

[50] Verdy A, Amarasekare P (2010) Alternative stable states in communities with intraguild predation. J theor Biol 262: 116–128. 19765596 10.1016/j.jtbi.2009.09.011

[51] Borrvall C, Ebenman B (2006) Early onset of secondary extinctions in ecological communities following the loss of top predators. Ecol Lett 9: 435–442. 16623729 10.1111/j.1461-0248.2006.00893.x

[52] Houlahan JE, Currie DJ, Cottenie K, Cumming GS, Ernest SKM, et al (2007) Compensatory dynamics are rare in natural ecological communities. Proc Natl Acad Sci USA 104: 3273–3277. 17360637 10.1073/pnas.0603798104 PMC1805590

[53] Mutshinda CM, O'Hara RB, Woiwod IP (2009) What drives community dynamics? Proceedings of the Royal Society B-Biological Sciences 276: 2923–2929. 10.1098/rspb.2009.0523 PMC2817208 19457887

[54] Jocque M, Vanschoenwinkel B, Brendonck L (2010) Freshwater rock pools: a review of habitat characteristics, faunal diversity and conservation value. Freshwater Biol 55: 1587–1602.

[55] Buenau KE, Rassweiler A, Nisbet RM (2007) The effects of landscape structure on space competition and alternative states. Ecol 88: 3022–3031. 10.1890/06-1850.1 18229837

[56] Jenkins DG, Ricklefs RE (2011) Biogeography and ecology: two views of one world. Phil Trans R Soc B Biol Sci 366: 2331–2335. 10.1098/rstb.2011.0064 PMC3130434 21768149

[57] Ricklefs RE, Jenkins DG (2011) Biogeography and ecology: towards the integration of two disciplines. Phil Trans R Soc B Biol Sci 366: 2438–2448. 10.1098/rstb.2011.0066 PMC3130436 21768158

[58] Weiher E, Freund D, Bunton T, Stefanski A, Lee T, et al (2011) Advances, challenges and a developing synthesis of ecological community assembly theory. Phil Trans R Soc B Biol Sci 366: 2403–2413. 10.1098/rstb.2011.0056 PMC3130429 21768155

